# Supplementary material for: Bacillus thuringiensis Bioinsecticides Induce Developmental Defects in Non-Target Drosophila melanogaster Larvae
Source: Insects. 2020 Oct 13;11(10):697. doi: 10.3390/insects11100697 (PMC7601982; doi:10.3390/insects11100697)
Supplement: Supplementary file 1 [file insects-11-00697-s001.pdf]

A **Evolution of intestinal flora in 5 days-old adults**

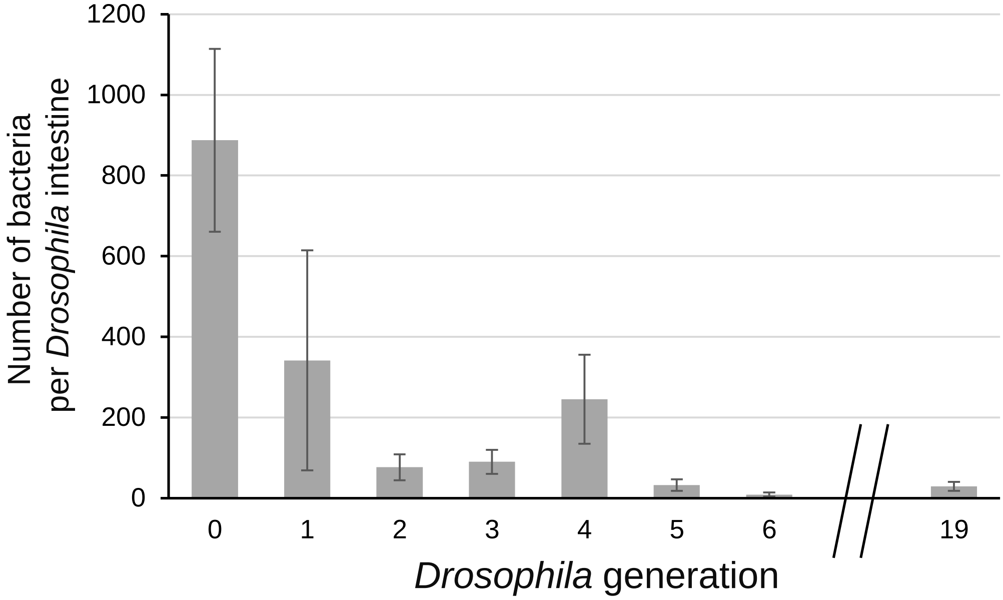

B **Intestinal flora in the fourth generation of adult flies**

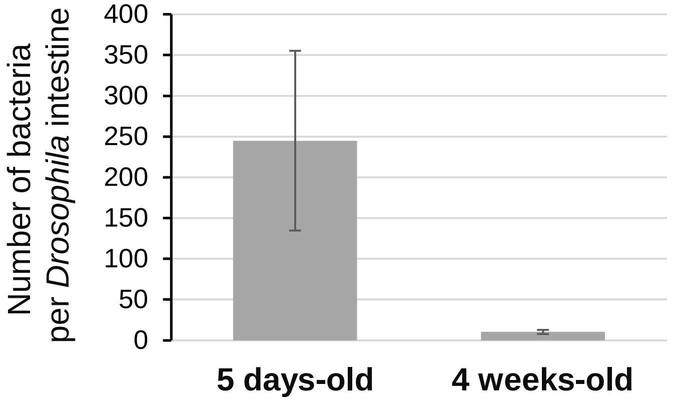

SupFigure 1

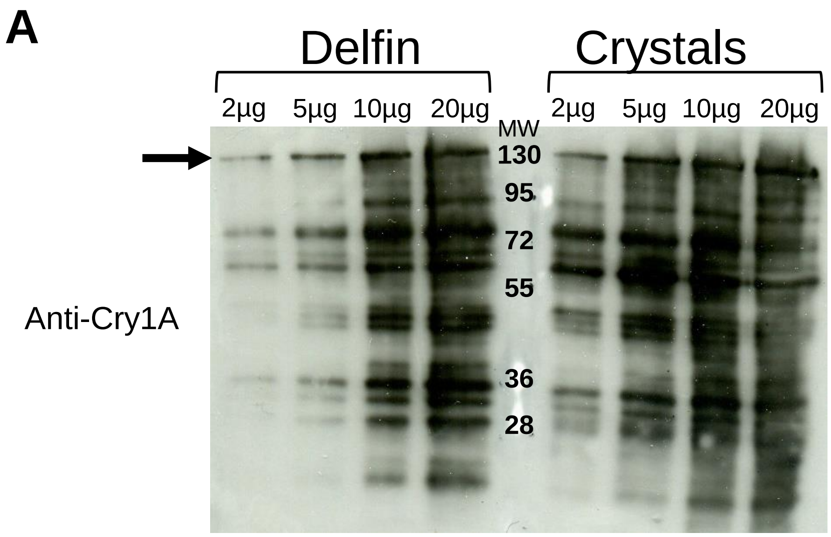
A
Delfin
Crystals
2µg
5µg
10µg
20µg
MW
2µg
5µg
10µg
20µg
130
95
72
55
36
28
Anti-Cry1A

SupFigure 2

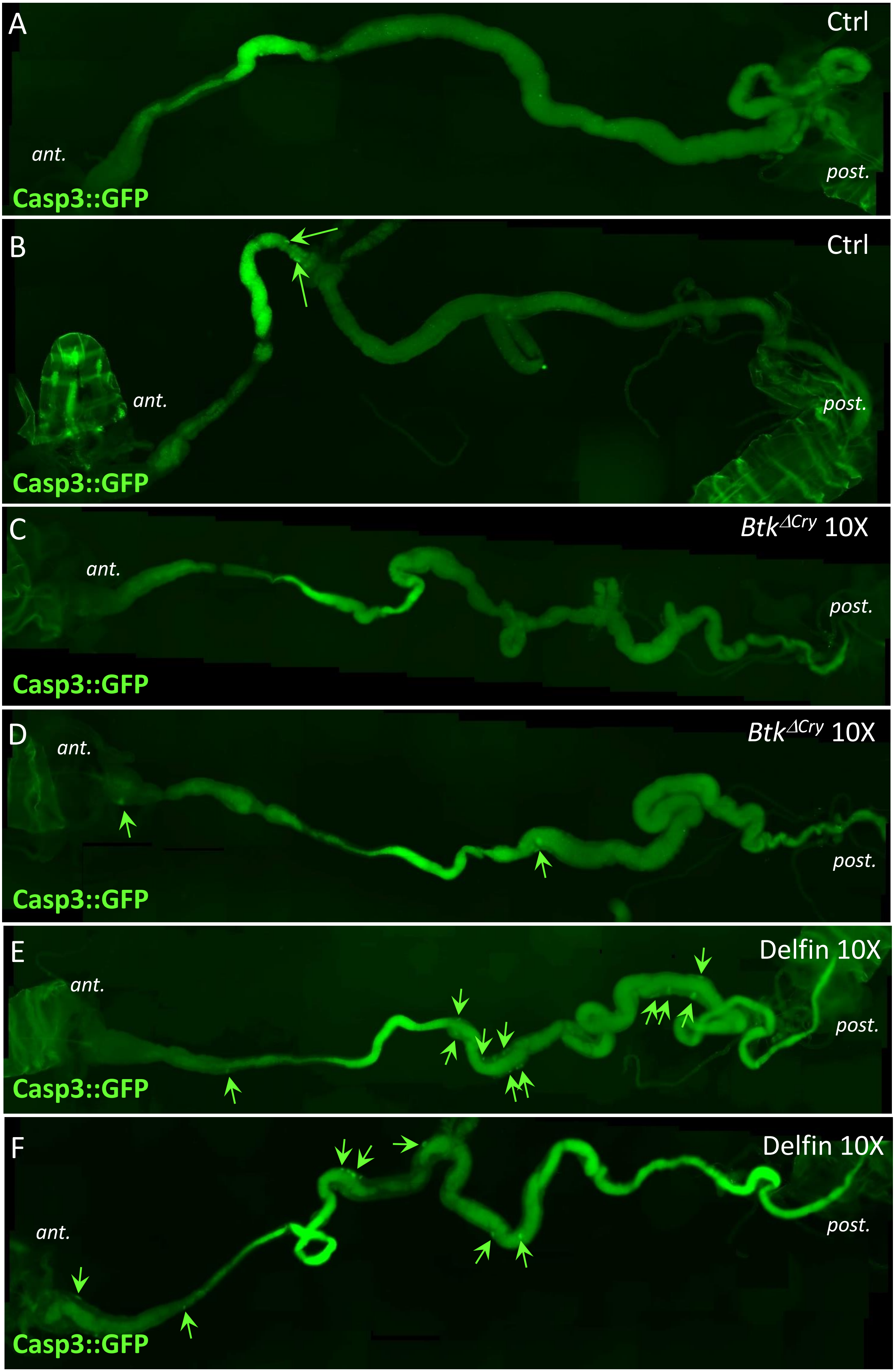
A
Ctrl
ant.
post.
Casp3::GFP
B
Ctrl
ant.
post.
Casp3::GFP
C
$Btk^{\Delta Cry}$ 10X
ant.
post.
Casp3::GFP
D
$Btk^{\Delta Cry}$ 10X
ant.
post.
Casp3::GFP
E
Delfin 10X
ant.
post.
Casp3::GFP
F
Delfin 10X
ant.
post.
Casp3::GFP

SupFigure 3

Supplemental legends of

Bacillus thuringiensis var. kurstaki induces developmental defects in non-target *Drosophila melanogaster* larvae

Marie-Paule Nawrot-Esposito, Aurélie Babin, Matthieu Pasco, Marylène Poirié, Jean-Luc Gatti and Armel Gallet

Supfigure 1: Commensal flora of Canton S adult *Drosophila melanogaster*
(A) Monitoring of whole intestinal commensal flora over 19 generations during the generation of the axenic strain. We considered that axenic flies were obtained at the 5th generation. We used axenic the flies from the 7th generation onwards to perform our experiments. **(B)** Intestinal commensal flora of young (5 days old) and aged (4 weeks old) adult flies of the 4th generation during the axenic establishment procedure. Error bars represent SEM.

Supfigure 2: Evaluation of the crystal amount
Western Blot (WB) using a Rabbit antiCry1A antibody (1/5000, Babin et al., 2019). Equal amounts (2, 5, 10 and 20µg) of Delfin (left part of the WB) and purified crystal (right part of the WB) were deposited on an SDS PAGE. We estimated that Crystals represent between 25% and 30% of the Delfin weight. The band at 130kDa corresponds to the Cry1A protoxins (upper arrow). MW: Molecular Weight in kiloDalton (kDa).

Supfigure 3: Caspase 3 activity in the L3 midgut
(A-F) *myo1A>Casp::GFP* larvae raised on control a medium (A nd B) or on a medium contaminated with $Btk^{\Delta Cry}$ 10X (C and D) or Delfin 10X (E and F). Arrows point enterocytes in which Caspase 3 is activated. Anterior is toward the left.
